# The prevalence of adolescent fatherhood and its associated factors in East African countries

**DOI:** 10.1186/s12889-024-19247-6

**Published:** 2024-06-27

**Authors:** Bewuketu Terefe, Enyew Getaneh Mekonen, Tadesse Tarik Tamir, Alebachew Ferede Zegeye, Belayneh Shetie Workneh, Masresha Asmare Techane

**Affiliations:** 1 Department of Community Health Nursing, School of Nursing, College of Medicine and Health Sciences, University of Gondar, PO Box: 196, Gondar, Ethiopia; 2 Department of Surgical Nursing, School of Nursing, College of Medicine and Health Sciences, University of Gondar, Gondar, Ethiopia; 3 Department of Pediatrics and Child Health Nursing, School of Nursing, College of Medicine and Health Sciences, University of Gondar, Gondar, Ethiopia; 4 Department of Medical Nursing, School of Nursing, College of Medicine and Health Sciences, University of Gondar, Gondar, Ethiopia; 5 Department of Emergency and Critical Care Nursing, School of Nursing, College of Medicine and Health Sciences, University of Gondar, Gondar, Ethiopia

**Keywords:** Adolescent fatherhood, Associated factors, Prevalence, East Africa

## Abstract

**Background:**

In developing nations, the phenomenon of adolescent fatherhood poses significant challenges, including increased risk of poverty, limited educational opportunities, and potential negative health outcomes for both the young fathers and their children. However, an overwhelming majority of research has concentrated on teenage motherhood. Adolescent fatherhood in poor nations has been the subject of little research. Few public health initiatives address adolescent fatherhood, in contrast to adolescent motherhood. Although there is currently more being done in industrialized nations to recognize adolescent fatherhood in clinical settings and the academic community. Undeveloped nations such as East Africa still have more problems that need to be resolved. Therefore, this study aimed to investigate the prevalence of and factors contributing to adolescent fatherhood in East Africa.

**Methods:**

Data from the Demographic and Health Surveys (DHS), collected between 2011 and 2022 in 12 East African nations, were used in this analysis. For a weighted sample of 36,316 male adolescents aged 15–24 years, we examined variables, as well as the prevalence of adolescent fatherhood. Univariate and multivariable logistic regression analyses were performed to identify candidate factors and significant explanatory variables associated with the outcome variable. The results are presented using adjusted odds ratios (AORs) at 95% confidence intervals (CIs). P values of ≤ 0.2 and < 0.05 were used to investigate statistically significant factors in the univariate and multivariable logistic regression analyses, respectively.

**Results:**

The overall prevalence of adolescent fatherhood was 11.15% (95% CI = 10.83,11.48) in East Africa. Age at first sex 20–24 years (AOR = 0.44, 95% CI:0.41,0.48), age–20–24 years old (AOR = 17.03,95% CI = 15.01,19.33), secondary/higher education (AOR = 0.57, 95% CI = 0.49,0.67), poor wealth (AOR = 2.27, 95% CI = 2.05,2.52), middle wealth (AOR = 1.70, 95% CI = 1.51,1.90), employed (AOR = 3.92, 95% CI = 3.40,4.54), utilized modern contraceptives (AOR = 0.75, 95% CI = 0.69,0.81), and female household heads (AOR = 0.43, 95% CI = 0.39,0.48) were associated with adolescent fatherhood.

**Conclusions:**

Adolescent fatherhood is more prevalent, in East Africa. These findings highlight the complexity of adolescent fatherhood and suggest that multiple factors, including socio-demographic characteristics and reproductive health behaviors, play a role in determining the likelihood of becoming an adolescent father. Understanding these associations can inform targeted interventions and policies aimed at reducing adolescent fatherhood rates and addressing the specific needs and challenges faced by young fathers in East Africa. Further research and interventions should focus on promoting education, economic opportunities, and access to modern contraceptives, while also addressing gender dynamics and social norms that contribute to adolescent fatherhood in the region.

## Introduction

A young man under the age of 24, who assumes parental responsibility for a child, regardless of the woman’s age, is said to be an adolescent father [1, 2]. This phenomenon is of significant interest due to its potential implications for both the adolescent father and the broader context of risky sexual behavior among adolescents. Hazardous sexual behaviors, including premarital sex, inconsistent condom use, and multiple sexual partners, have been identified as contributing factors to the increasing prevalence of risky sexual behavior among adolescents worldwide [3–5]. Unwanted pregnancies, early parenthood, early fatherhood, and STIs/HIV are only a few outcomes of these behaviors [1, 5–7]. Adolescent fatherhood and the factors that impact it, particularly in sub-Saharan Africa, have received less attention than teenage pregnancies and child motherhood. In this area, men’s sexuality is generally permeated by unrestrained acts of sex outside of marriage, where masculinity is supreme [8–10]. Risky sexual behavior impacts partners and children beyond individuals. Adolescent fathers contribute to unplanned pregnancies, which can lead to absent fathers [11–13]. Children without active fathers face developmental challenges like poverty, dropout, addiction, and incarceration [11, 14, 15]. Adolescent mothers may struggle with single parenthood, affecting education and their child’s well-being [12, 13]. Adverse outcomes like reduced education perpetuate disadvantage, affecting children’s development negatively [12, 14, 15].

More than a billion adolescents live in poor nations, where they are physically able to procreate, but are too young to be responsible for a partner and children [1]. Contrary to a comprehensive study on parenting and female fertility, adolescent fatherhoods are largely absent from public statistics, and no study has offered information on the ideal age for fatherhood [1]. Adolescents worldwide are increasingly showing signs of high-risk sexual behavior and sexual knowledge. Over 20% of adolescents in sub-Saharan Africa who have ever engaged in sexual activity also have several partners [16, 17]. According to a study conducted in Zimbabwe, out of the 40 adolescent fathers surveyed, 55% had already had sex, and this evidence shows that a substantial portion of adolescents have been sexually active [3]. According to another study, 70% of the 145 teenagers who engaged in premarital sex were male [6]. Furthermore, a reports from Nigeria, about 35% of the 200 adolescents questioned had at least fathered a child or impregnated a female [1, 7]. More adolescent fatherhood today than in the 20th century boast about their sexual prowess and work to improve their sex skills [3].

Adolescents, both males and females, especially males, are engaging in premarital sexual activity at earlier ages [18, 19]. Additionally, men are dominant in patriarchal and patrilineal systems in Africa [20], and their choices, behaviors, and supports are similar to the outcomes of women’s sexual activity [7, 20]. Adolescent fatherhood is significantly influenced by patriarchal norms that prioritize men as providers, perpetuating unequal power dynamics and shaping decision-making and support [7, 21, 22]. The pressure to conform to traditional gender roles affects behaviors and expectations, including the division of household labor and child-rearing responsibilities [7, 22, 23]. Adolescent fatherhood intersects with patriarchal norms, reflecting and reinforcing traditional gender roles, power dynamics, and societal expectations [22–24]. Recognizing and understanding these dynamics is crucial for addressing the impact of patriarchal norms on adolescent fathers, their partners, and children, and for promoting more equitable and supportive family structures (7, 24). Therefore, if the male aspect is not given sufficient consideration, the answers to a variety of sexuality issues may only be a mirage. While there are many studies on teen and teenage pregnancy and significant funding devoted to prevention methods, there is comparatively little discussion of concerns connected to adolescent fatherhood among researchers and in terms of public initiatives [7, 23].

This study aims to significantly contribute to the limited body of literature on adolescent fatherhood in East Africa by addressing population gaps, bridging critical knowledge gaps, providing robust empirical evidence, and filling substantial voids in the existing literature in the region. Moreover, obtaining a deeper understanding of the factors that influence adolescents’ choices to engage in adolescent fatherhood will not only aid in the creation of intervention packages that are more widely embraced but also strengthen efforts to safeguard the rights of adolescents in the region. By utilizing data from recent demographic and health surveys, this study rigorously examines the frequency and underlying factors influencing adolescent fatherhood in East Africa. The findings of this study have the potential to yield valuable insights for policymakers, effectively fill the void resulting from the limited attention given to adolescent fatherhood, and guide governmental and non-governmental organizations in the development of targeted interventions to mitigate the prevalence and adverse consequences of adolescent fatherhood. Therefore, the primary objective of this study is to comprehensively understand the prevalence and determinants of adolescent fatherhood in East Africa, thereby shedding new light on this underexplored area of research.

## Methods

### Data source and study population

The information is derived from a ten-year (2011–2022) span of the most recent standard Demographic and Health Survey (DHS) data, specifically the men’s record (MR) dataset, for East African countries, namely Burundi, Comoros, Ethiopia, Kenya, Madagascar, Malawi, Mozambique, Rwanda, Tanzania, Uganda, Zambia, and Zimbabwe. Despite the fact that the DHS was carried out in 14 eastern nations between 2011 and 2022, only about 12 of them were taken into consideration for our analysis because the remaining surveys lacked data on the outcome variable (Table 1). The final analysis used a total weighted sample of 36,316 and an unweighted sample of 36,560 male adolescents who had been interviewed for adolescent fathers after the data from each nation had been incorporated.

**Table 1:** Countries, sample size, and survey year of demographic and health surveys included in the analysis for 12 East African countries

Country	Survey year	Sample size(weighted)	Frequency(weighted)
Burundi	2016/17	2,782	7.66
Ethiopia	2016	4,455	12.27
Kenya	2022	5,579	15.36
Comoros	2012	827	2.28
Madagascar	2021	3,428	9.44
Malawi	2016/17	3,226	8.88
Mozambique	2011	1,519	4.18
Rwanda	2019/20	2,486	6.84
Tanzania	2022	1,508	4.15
Uganda	2016	2,238	6.16
Zambia	2018	4,813	13.25
Zimbabwe	2015	3,456	9.52

Demographic and Health Surveys (DHS) utilize conventional census frames and employ a stratified, two-stage cluster sampling technique. Urban and rural areas within each administrative geographic region were normally separated from the DHS samples. Enumeration areas (EAs) within each stratum were selected in the initial sampling, with a probability proportional to their size. In the second stage, the systematic sampling approach selects a predetermined number of residences in the designated EAs. After the household listing, a certain number of households from inside the selected cluster are chosen using equal probability systematic sampling [25].

### Study variables

The dependent variable in this study was adolescent fatherhood. Adolescent fatherhood was defined as having at least one child before the age of 24. It was generated from the DHS variables “the number of children ever fathered”. This was recorded as a binary variable, with “1” indicating the respondent had at least one child, and “0” indicating they had none. The following independent variables were taken into consideration for this study: respondent age, age at first sex, educational level, marital status, wealth status, sex of household head, employment status, age at first sex, media exposure, knowledge of modern contraceptives, and types of residence were all thought to be potential factors for the outcome variable (Table 2).

**Table 2:** Sociodemographic characteristics of adolescent fatherhood in East Africa, based on 2011–2022 DHS data (unweighted *n* = 36,560, and weighted *n* = 36,316)

Variables	Weighted sample	Weighted frequency
Respondent age in years		
15–19	21,225	58.45
20–24	15,091	41.55
Educational attainment		
Not educated	1,711	4.71
Primary	16,391	45.13
Secondary/higher	18,214	50.15
Age first sex		
15–19	12,769	35.16
20–24	23,547	64.84
Ever been married/union(*n* = 16,918)		
No	15,439	91.26
Yes	1,480	8.74
Wealth index		
Poor	12,226	33.67
Middle	7,553	20.80
Rich	16,537	45.54
Residence		
Urban	10,052	27.68
Rural	26,264	72.32
Employment		
No	11,216	30.88
Yes	25,100	69.12
Contraceptive usage		
No	9,164	25.23
Yes	27,152	74.77
Knowledge of modern contraceptive		
No	1,016	2.80
Yes	35,300	97.20
Mass media exposure		
No	13,778	37.94
Yes	22,538	62.06
Sex of household head		
Male	26,355	72.57
Female	9,961	27.43

### Data management and statistical analysis

The study variables were extracted, cleaned, and recoded using STATA version 17. In any statistical analysis, the data were weighted using sample weights to account for the differential probability of selection resulting from the sampling method used to collect the DHS data. As a result, the representativeness of the survey results was guaranteed. For the univariate analysis, we used logistic regression to examine the association between each individual independent variable and the binary dependent variable of adolescent fatherhood. The results of this univariate logistic regression analysis, including the crude odds ratios, 95% confidence intervals, and p-values, are presented in Table 3. The Adjusted Odds Ratio (AOR) with 95% confidence interval for the multivariable logistic model’s best fit was determined to identify the contributing factors to adolescent fatherhood. Descriptive categorical data were compiled using descriptive studies, such as frequency count and proportion. To examine multicollinearity among independent variables, a logistic model was fitted using the variance inflation factor. The Hosmer-Lemeshow test was used to evaluate the final regression model’s overall fitness. The statistical significance level of the final model was set at *P* < 0.05.

**Table 3:** Shows the factors crude odds ratios and their corresponding p value in the univariate logistic regression analysis among adolescents in East Africa

Variables	COR 95% CI	*P* value
Age	23.87(21.12,26.97)	0.0001
Educational attainment	0.67(0.64,0.71)	0.0001
Age first sex	0.22(0.21,0.24)	0.0001
Wealth index	0.69(0.67,0.72)	0.0001
Residence	1.51(1.39,1.63)	0.0001
Employment	9.76(8.51,11.19)	0.0001
Contraceptive usage	2.66(2.48,2.84)	0.0001
Mass media exposure	0.98(0.92,1.05)	0.0643
Sex of household head	0.39(0.36,0.43)	0.0001

Because the data could be hierarchical, we examined it for the assumption of multilevel model analysis using the intra-class correlation (ICC) coefficient, but it was only 1.4%, which fell short of the required minimum to employ the multilevel model. It was decided that conventional logistic regression was preferred as a consequence. Furthermore, before proceeding to the analysis, each dependent variable was assessed for its variance, inflation factors, and tolerances. The mean VIF in this study was 1.09.

## Results

### Sociodemographic characteristics of the study participants

More than half 21,225 (58.45%) of the study participants were between 15 and 19 years old. Regarding marital status, almost all of them 15,439 (91.26%) were never married. With respect to the place of residence types, 26,264 (772.32%), educational status 18,214 (50.15%), and wealth index 16,537 (45.54%) of adolescents were from rural areas, completed secondary/higher educational level, rich households respectively. About 23,547 (64.84%) had started their first sex between 20 and 24 years. The majority 25,100(69.12%), are employed. Regarding knowledge and usage of modern contraceptives about 35,300 (97.20%), and 27,152(74.77%) of them were knowledgeable, and used modern contraceptives respectively. About 22,538(62.06%), of adolescents had media exposure (Table 2).

### Prevalence of adolescent fatherhood in East Africa

**Fig. 1:**
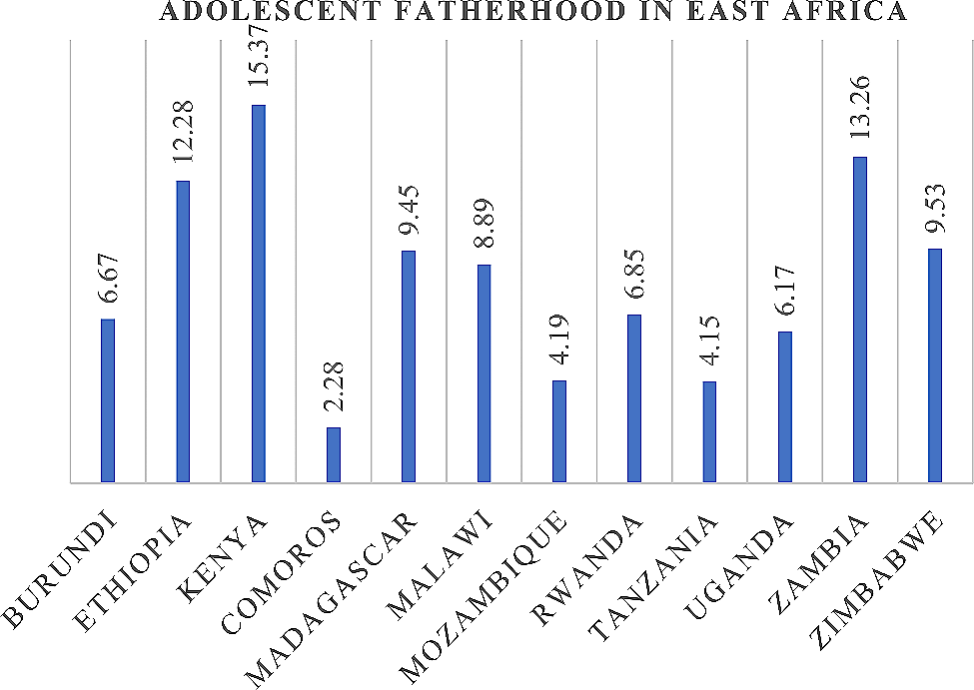
The prevalence of adolescent fatherhood in East Africa

In this study the overall polled prevalence of adolescent fatherhood was 11.15% (95% CI = 10.83,11.48) among adolescents aged from 15 to 24 years old in 12 East African countries. The highest adolescent fatherhood was prevailed in Kenya (13.37%), and the lowest was from Comoros (2.28%) (Fig. 1).

### Factors associated with adolescent fatherhood among adolescents in East Africa

The odds of adolescent fatherhood were decreased by 56% (AOR = 0.44, 95% CI:0.41,0.48) among adolescents who had started their first sexual intercourse at the age of 20–24 years of as compared to 15–19 years old adolescents. In addition, as compared to 15-19-year-old adolescents, those age between 20 and 24 years old showed a higher (AOR = 17.03,95% CI = 15.01,19.33) frequency of becoming an adolescent father. Compared to uneducated adolescents, those who had finished their secondary/higher educational attainment showed a lower odd of having a child during adolescence (AOR = 0.57, 95% CI = 0.49–0.67). Conversely, regarding the household wealth index, adolescents who came from poor and middle households showed a higher likelihood of being a father than those who came from the rich household wealth index by the odds of (AOR = 2.27, 95% CI = 2.05,2.52) and (AOR = 1.70, 95% CI = 1.51,1.90) times more, respectively. Being employed showed a statistically significant association with adolescent fatherhood (AOR = 3.92, 95% CI = 3.40,4.54) more times than unemployed participants. Adolescents who have utilized modern contraceptives have shown (AOR = 0.75, 95% CI = 0.69,0.81) lower likelihood of having a child during adolescence. Similarly, adolescents from female household heads (AOR = 0.43, 95% CI = 0.39,0.48) were lower than those from male household heads (Table 4).

**Table 4:** Multivariable logistic regression analysis for the assessment of determinants of adolescent fatherhood in East Africa, 2011–2022

Fathered children	No, *n* (%)	Yes, *n* (%)	COR (95% CI)	AOR (95% CI)
Variables				
Respondent age				
15–19	20,925(98.58)	301(1.42)	1	1
20–24	11,342(75.16)	3,749(24.84)	23.86(21.12,26.97)	17.03(15.01,19.33) *
Age at first sex				
15–19	10,044(78.65)	2,726(21.35)	1	1
20–24	22,223(94.38)	1,324(5.62)	0.23(0.21,0.24)	0.44 (0.41,0.48) *
Education status				
Not educated	1,377(80.49)	334(19.51)	1	**1**
Primary	14,333(87.44)	2,058(12.56)	0.62(0.55,0.71)	0.91(0.78,1.06)
Secondary/higher	16,557(90.90)	1,658(90.90)	0.43(0.38,0.49)	0.57(0.49,0.67) *
Wealth				
Poor	10,342(84.59)	1,884(15.41)	2.10(1.95,2.26)	2.27(2.05,2.52) *
Middle	6,697(88.66)	857(11.34)	1.57(1.43,1.73)	1.70(1.51,1.90) *
Rich	15,228(92.08)	1,309(7.92)	1	1
Residence				
Urban	9,153(91.06)	899(8.94)	1	1
Rural	23,113(88.00)	3,151(12.00)	1.51(1.39,1.63)	1.09 (0.92,1.13)
Employment				
No	11,012(98.18)	204(1.82)	1	1
Yes	21,255(84.68)	3,846(15.32)	9.76(8.51,11.19)	3.92 (3.40,4.54) *
Contraceptive usage				
No	7,357(80.28)	1,807(19.72)	1	1
Yes	24,910(91.74)	2,242(8.26)	0.38(0.35,0.41)	0.75 (0.69,0.81) *
Mass media exposure				
No	12,211(88.63)	1,567(11.37)	1	1
Yes	20,055(88.98)	2,483(11.02)	0.98(0.92,1.05)	0.97 (0.90,1.05)
Sex of household head				
Male	22,840(86.66)	3,516(13.34)	1	1
Female	9,427(94.64)	534(5.36)	0.39(0.36,0.43)	0.43 (0.39,0.48) *

Where * indicates significant variables at *p* value of < 0.05 in the final model

## Discussion

In this study, we examined the factors of adolescent fatherhood in different countries using DHS data from East African countries. Our research highlights the significance of previously identified factors that influence adolescent fatherhood, as found in literature from different nations. Age, age at first sex, education, money, employment position, sex of the household head, and use of contemporary contraceptives were statistically significant factors in this study’s analysis of the relationship between adolescent fatherhood.

According to the study, individuals who had their sexual debut at a later age compared to their peers had a lower odd of becoming fathers during adolescence, which is consistent with research from Ethiopia and Brazil [26, 27]. Because early sex initiation is linked to a higher chance of early or undesired births, it raises the possibility of adolescent fatherhood [28]. Early sexual activity is also linked to a higher chance of having several sexual partners over one’s lifetime, unprotected sex, and substance abuse [29, 30]. The timing of first sexual encounters has been found to be influenced by parental involvement and family dynamics, with permissive and negligent parenting styles being linked to increased risk of sex activity and hazardous sex [30]. On the contrary, adolescents today are also exposed to media and technology, which can have an impact on their decision-making [31].

The results indicate that adolescent fatherhood between the ages of 20 and 24 are more likely to become fathers than those between the ages of 15 and 19. The following elements can play a role: older age during adolescence was found to be significantly associated with higher odds of becoming a father during the teenage years. Studies have shown that as adolescents age, they are more likely to engage in sexual activity and experience early fatherhood [26, 32, 33]. Many adolescent fathers start committed relationships with the mother of their child around the time of the child’s birth, although many of these relationships dissolve before the child turns one [33]. This study aims to build on prior research exploring how social norms and perceptions surrounding teenage pregnancy can impact the relationships and parenting experiences of adolescent parents. The core focus is to examine the relationship between partnership instability among young parents and child well-being, specifically investigating the timing of relationship formation and dissolution, as well as the marital/relationship status of the young parents and reasons for non-marital status like teenage pregnancy [34–36]. The goal is to gain a deeper understanding of how the social context, attitudes, and real-world relationship dynamics of adolescent parents may contribute to the challenges and consequences for their children. Compared to their white counterparts, Hispanic and black youths are more than twice as likely to become fathers [33]. Adolescent fathers are often unmarried at the time of conception and childbirth, resulting in their exclusion from participating in the birth and early care of their infants. However, in some cases, adolescent fathers may be married and have paid the bride price, allowing them to stay together with their partners and be present for the birth. This is typically the outcome of the strong societal stigma associated with teenage pregnancy and childbirth [32]. Income and academic success: Having children when still a teen is associated with a number of characteristics, including low income and subpar academic performance [26]. It is significant to remember that having children when still a teen might have negative effects on the guy, his partner, and his children [32]. Compared to their classmates, children with highly involved fathers and high-risk mothers exhibit less behavioral issues [33].

Additionally, we discovered that adolescent fathers from families headed by women had a decreased chance of becoming fathers at a younger age. This may be because females interact and communicate deeply with their children and are more knowledgeable about contraceptive options and how to use them effectively, all of which help to prevent adolescent fatherhood [26, 32]. According to the study, maternal absence during adolescence was more than three times as strongly associated with early fatherhood as paternal absence [15]. Compared to fathers, mothers in lower and middle income countries have been shown to have a larger part in raising their children and spend more time giving them cross-cultural care and support [37], which perhaps explains why their absence can have a more significant impact on their children [38].

Prior studies have repeatedly shown that poverty and low educational attainment are significant predictors of early fatherhood among adolescents [12, 15]. Adolescents with higher levels of education and from wealthier households have greater knowledge of sexual and reproductive health, delay the age at first sexual activity, are less likely to be married, have more household resources, and have higher future aspirations. Also, these adolescents, even if they are sexually active, often protect themselves when having sexual intercourse [39, 40]. Due to increased stress at home, poor families may not have access to sufficient resources, and family income inequality increases the likelihood of neglect, criminal activity, and physical abuse. It’s also important to note that men with college degrees are much more likely than men without college degrees to put off starting a family [41]. Fathers who are younger, less educated, poorer, and non-white seem to be more likely to be single parents [42].

For women who are young, single, students, or in unstable relationships, using modern contraceptives to delay their first pregnancy is often acceptable [43]. Early fatherhood can be decreased by delaying the first delivery. By avoiding unplanned pregnancies, which are more likely to result in abortion, increased access to contraception can help decrease the expensive and frequently dangerous reliance on abortion [44]. By doing this, fewer unwanted pregnancies that result in early fatherhood may occur. The two factors that have the biggest influence on lowering a nation’s fertility rate are widespread use of contraceptives and, to a lesser extent, ensuring that females complete at least 14 years of education [45, 46]. The number of unplanned pregnancies and early fatherhood can be decreased by lower fertility rates.

The fact that this study was based on weighted, nationally representative data with a substantial sample size provides strength. Additionally, being derived from a national survey, the study has the potential to offer policymakers and program planners valuable information for developing effective intervention strategies. Implementing interventions, such as comprehensive sex education programs, ensuring access to affordable and youth-friendly reproductive health services, and establishing social support networks and mentoring programs for adolescent fathers, can assist them in navigating the challenges they face at both national and regional levels. This study was constrained by the fact that the DHS survey relied on respondents’ self-reports, and might have been subject to recollection bias. Additionally, even though these variables are significant factors that affect adolescent fatherhood, they were not included in the DHS. These variables included community attitudes toward marriage, norms, values, and religious beliefs regarding marriage and fatherhood. Finally, the study’s findings are based on data collected at a single point in time, and therefore, it is not possible to determine whether the identified associations between factors and adolescent fatherhood are causal or simply correlational. Longitudinal or experimental study designs would be better suited for establishing causal relationships and understanding the temporal sequence of events. While the study provides valuable insights into the prevalence and factors associated with adolescent fatherhood in East Africa, caution should be exercised in interpreting the findings as causal relationships cannot be inferred from the data.

## Conclusions

East Africa had a slightly higher average prevalence among adolescent fathers. The outcome variable was significantly associated with the respondent’s age, age at first sex, employment status, wealth index, education, sex of the household head, and use of modern contraceptives. Our study provides some estimates of these correlations in the context of a developing nation and adds to the scant worldwide literature on contemporary factors and the effects of adolescent fatherhood. To comprehensively address the underlying risk factors and postpone the onset of early parenthood in adolescent fathers, it is crucial to combine sexual and reproductive health campaigns with broader prevention strategies, such as quality secondary education, parental engagement, strengthening family planning for youth-friendly services, and poverty alleviation programs and policies.

### Policy implications and the way forward

The findings suggest that specific interventions should be designed to address the higher prevalence of adolescent fatherhood in East Africa. Policymakers can focus on implementing targeted programs and policies that address the unique challenges faced by adolescent fathers in the region. The association between the outcome variable and factors such as age at first sex, education, and use of modern contraceptives highlights the importance of comprehensive sex education. Policymakers can prioritize the implementation of evidence-based sex education programs that provide accurate information about reproductive health, contraception, and responsible sexual behavior. Moreover, the study’s association between employment status and adolescent fatherhood suggests the need for policies that promote economic empowerment among young men. Policymakers can focus on creating vocational training programs, job opportunities, and supportive policies that enable young men to secure stable employment and financial independence. Again, the finding related to the sex of the household head underscores the importance of gender equality in addressing adolescent fatherhood. Policymakers can work towards promoting gender equality and challenging traditional gender norms that contribute to early fatherhood. This can involve initiatives that promote equal opportunities for education and employment for both genders. Finally, the association between the outcome variable and the wealth index highlights the need to ensure equitable access to affordable and youth-friendly reproductive health services. Policymakers can invest in improving the accessibility and affordability of contraceptives, healthcare services, and counseling for young men, particularly those from disadvantaged backgrounds.

## Data Availability

All data concerning this study are accommodated and presented in this document. The detailed data set can be freely accessible from the www.dhsprogram.comwebsite.
